# Role of EPAC1 Signalosomes in Cell Fate: Friends or Foes?

**DOI:** 10.3390/cells9091954

**Published:** 2020-08-25

**Authors:** Karina Formoso, Frank Lezoualc’h, Jeanne Mialet-Perez

**Affiliations:** INSERM UMR-1048, Institute of Metabolic and Cardiovascular Diseases, and Université de Toulouse III-Paul Sabatier, 31432 Toulouse, France; karina.formoso@inserm.fr (K.F.); Jeanne.perez@inserm.fr (J.M.-P.)

**Keywords:** EPAC1, cAMP, signalosomes, hypertrophy, cell death

## Abstract

The compartmentation of signaling processes is accomplished by the assembly of protein complexes called signalosomes. These signaling platforms colocalize enzymes, substrates, and anchoring proteins into specific subcellular compartments. Exchange protein directly activated by cAMP 1 (EPAC1) is an effector of the second messenger, 3′,5′-cyclic adenosine monophosphate (cAMP) that is associated with multiple roles in several pathologies including cardiac diseases. Both EPAC1 intracellular localization and molecular partners are key players in the regulation of cell fate, which may have important therapeutic potential. In this review, we summarize the recent findings on EPAC1 structure, regulation, and pharmacology. We describe the importance of EPAC1 subcellular distribution in its biological action, paying special attention to its nuclear localization and mechanism of action leading to cardiomyocyte hypertrophy. In addition, we discuss the role of mitochondrial EPAC1 in the regulation of cell death. Depending on the cell type and stress condition, we present evidence that supports either a protective or detrimental role of EPAC1 activation.

## 1. Introduction

3′,5′-Cyclic adenosine monophosphate (cAMP) is a universal second messenger that regulates a wide spectrum of signaling pathways in cells [1,2]. In mammals, cAMP is synthesized from ATP by a family of enzymes named adenylate cyclases (ACs). The AC family is composed of 9 membrane-bound members (AC1–9) that are activated by G protein-coupled receptors (GPCRs) linked to stimulatory G proteins (Gs). The tenth member of the family is the soluble isoform AC10, more commonly known as sAC [3]. The activation of different signaling pathways in one cell by cAMP is not a casual but a rather complex and controlled mechanism that involves specific machinery. Indeed, compartmentation of cAMP involves various proteins including the cAMP-degrading enzymes, phosphodiesterases (PDEs), and scaffolding proteins such as A-kinase anchoring proteins (AKAPs) that sequester cAMP signalosome into specific places within the cell [3]. In addition, the functional effect of cAMP is achieved by different effectors that act alone or in concert with other proteins. Among them, the protein kinase A (PKA) is the one that has been described first and the one more thoroughly studied [4]. However, other cAMP effectors have been identified including the exchange proteins directly activated by cAMP (EPACs) [5,6,7].

The two EPAC isoforms, EPAC1 and EPAC2, are guanine nucleotide exchange factors (GEFs) that activate the Ras-like small GTPases, Rap1, and Rap2. EPAC1 is ubiquitously expressed and its potential involvement in central and peripheral diseases has been largely studied in recent years. For instance, EPAC1 has been linked to Alzheimer’s disease, depression, and retinal neurodegeneration [8,9]. EPAC1 is also involved in migration, proliferation, and apoptosis in several types of cancers [9,10,11,12]. The role of EPAC1 in cardiac pathologies such as heart failure and arrhythmias has been characterized using EPAC1 transgenic mouse models, although it remains a matter of discussion [13,14]. Consistent with cAMP compartmentation, EPAC1 is spatially and temporally regulated by scaffolding proteins and its effectors interconnect with other signaling pathways. Due to its multidomain structure, EPAC1 expresses within different subcellular compartments such as the nucleus, the cytosol, the mitochondria, and plasma membranes and its localization may vary depending on the cell type and the cell cycle phase [15]. Unravelling the mechanisms underlying EPAC1 subcellular distribution and its functional relevance in distinct pathologies will have an important impact on determining EPAC1’s potential role in therapy. After a brief description of the gene and the protein structure of EPAC1, this review will summarize the latest pharmacological tools developed to modulate EPAC1 activity. In addition, we will detail recent findings on EPAC1 compartmentation paying special attention to the functions exerted by the protein at the nuclei and mitochondria and their beneficial or detrimental effects on cell destiny. Given the presence of extensive bibliography in the field, we will emphasize the importance of these processes in the context of cardiac pathophysiology.

## 2. Gene, Expression, and Structure

EPAC1 is encoded by the Rap guanine nucleotide exchange factor 3 (*RAPGEF3*) gene that possesses three and four validated isoforms in humans (www.ncbi.nlm.nih.gov/gene/10411) and mice (www.ncbi.nlm.nih.gov/gene/223864), respectively. The Rap guanine nucleotide exchange factor 4 (*RAPGEF4*) gene codes for five EPAC2 validated isoforms in mice (https://www.ncbi.nlm.nih.gov/gene/56508) and eighteen EPAC2 variants in humans (https://www.ncbi.nlm.nih.gov/gene/11069). RAPGEF3and *RAPGEF4* genes are located in different chromosomes but share high sequence homology and functional similarities. Their difference lies mostly in their pattern of expression. In this sense, *EPAC1* expression is rather ubiquitous and is found with high expression levels in the heart and kidney. Instead, *EPAC2* expression is much more restricted being mainly expressed in the central nervous system and endocrine tissues including the pancreas [5,6,9]. Though the regulation of *EPAC1* expression has not been fully described, work from Lai and collaborators [16] showed that binding of hypoxia-inducible factor 1α (HIF-1α) to the hypoxia-responsive element of the *EPAC1* promoter might induce its transcription in mouse primary cortical cultures subjected to hypoxia. In addition, recent studies found that both mRNA and protein expression of EPAC1 are dependent on the TEAD transcription factor and the H3K27 acetylation (Histone 3 lysine 2) of the promoter of *EPAC1*. Upon cAMP-elevating stimuli, the inhibition of YAP/TAZ TEAD and HDAC1/3-mediated H3K27 deacetylation leads to decreased EPAC1 expression in cardiac fibroblasts. These results suggest that there might be a negative feedback loop on EPAC1 expression upon cAMP generation [17].

**Figure 1:**
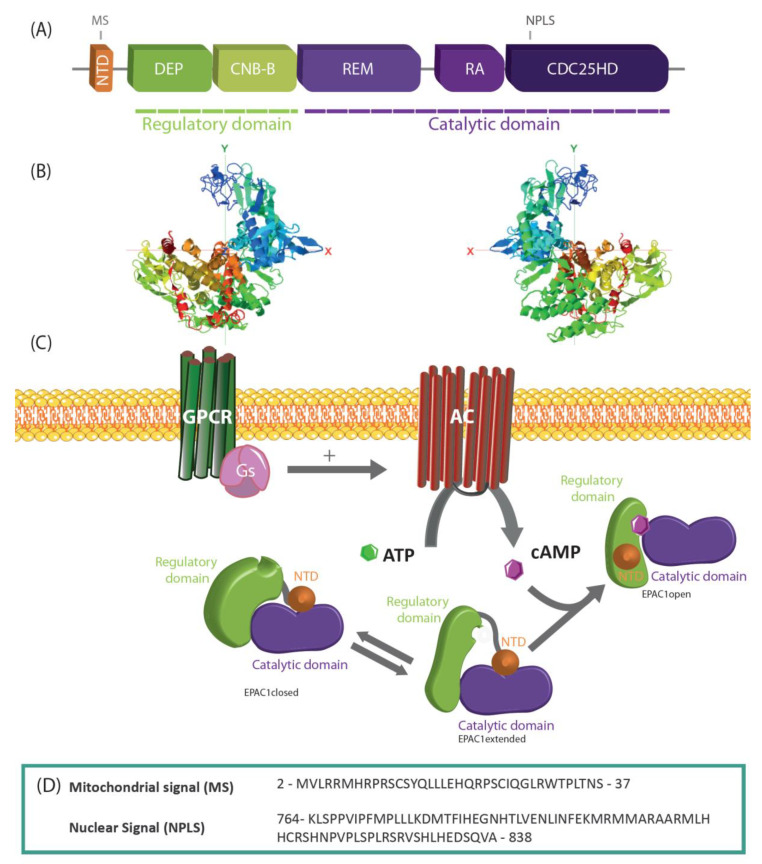
EPAC1 structure and activation. (**A**) Descriptive graph of EPAC1 domains. NTD, N-terminal domains (orange); Regulatory domain (green): DEP, disheveled-Egl10-pleckstrin domain; CNB-B, cyclic nucleotide-binding domain B; catalytic domain (purple): RA, RAS association domain; REM, RAS exchange motif; CDC25-HD, cell division & cycle 25 homology domain. (**B**) I-TASSER generated three-dimensional model of EPAC1 viewed from two different angles [23,24] (**C**) The conformational states of EPAC1 upon activation are depicted as follows: EPAC1closed state (inactive), EPAC1extended state (inactive), and EPAC1open state (active). (**D**) EPAC1 sequences corresponding to the mitochondrial and nuclear localization signals.

EPAC1 is a monomeric protein of approximately 100 KDa that contains a regulatory region and a catalytic region (Figure 1A,B). The regulatory region located in the N-terminal part of EPAC1 is composed of an N-terminal domain (NTD), the disheveled-Egl10-pleckstrin (DEP) domain, which ensures EPAC1 localization at the plasma membrane and a cyclic nucleotide-binding domain (CNB-B) that regulates EPAC1 GEF activity [18]. Both PKA and EPAC1 have a CNBD domain. It was initially proposed that the concentration of cAMP required to activate EPACs was 10 fold higher than to activate PKA [19]. However, further in vitro studies on the PKA in the holoenzyme complex and EPAC1 showed similar affinities for cAMP binding [20]. Of note, EPAC2 harbors a second CNB domain (named CNB-A) which binds cAMP with low affinity. CNB-A is not required for EPAC2 activation by cAMP but is involved in its subcellular localization [21]. The C-terminal part of the EPAC1 protein contains the catalytic region and possesses 3 defined domains, namely the Ras association domain (RA), the RAS exchange motif (REM), that is involved in the stabilization of the active conformation of EPAC1 and a cell division and cycle 25 homology domain (CDC25-HD). The latter is responsible for the guanine nucleotide exchange for Rap GTPases, Rap1, and Rap2 and possesses a nuclear targeting signal whose importance will be discussed below [9,22].

Most of the structural analyses on EPAC1 derive from EPAC2, given that purified recombinant EPAC1 protein is resistant to crystallographic study. The X-ray crystal structure studies have revealed that EPAC exists in two conformational states: In its inactive form (apo-EPAC), the regulatory region blocks the catalytic domain thereby, inhibiting the interaction of EPAC with its effector, Rap. Binding of cAMP induces a conformational change that allows the CDC25-HD catalytic region to interact with its Rap effector [25,26]. More recently, White and colleagues [27] using Small-Angle X-ray Scattering (SAXS) have shown that EPAC1 exists in three conformational states, two of which are in equilibrium in the absence of cAMP (EPAC1-closed and EPAC1-extended). In the extended conformation, the conformation of CNB is similar to a cAMP-bound like conformation but without the cAMP-binding pocket covered by a lid. Upon cAMP binding to the intermediate (EPAC1 extended) state, the regulatory NTD/DEP/CNB domain is locked away from the catalytic domains (Figure 1C) [27].

## 3. EPAC1 Pharmacological Modulators

Given the role of EPAC1 in disease development, there is a growing interest in identifying selective pharmacological molecules to modulate its activity. Several compounds specifically targeting EPAC1 have been described in recent reviews so we will describe briefly the last advances in the area [14,28]. The EPAC1 agonists are classified into nucleotide-like activators and non-cyclic-nucleotide activators. The cAMP analog, 8-CPT (8-(4-chloro-phenylthio)-2′-O-methyladeno- sine-3′,5′-cAMP) was the first EPAC1 agonist identified and it has been largely used to uncover EPAC1 biological action in cultured cells [19]. The 8-CPT compound preferentially activates EPAC1 over EPAC2 and is 30 to 100 times less potent than cAMP to activate PKA [19,29,30] but as 8-CPT displays a poor membrane permeability, an acetoxymethyl ester derivative named 8-CPT-AM was developed [31]. This chemical modification significantly improved the efficiency of the mother molecule, 8-CPT [31]. However, 8-CPT and 8-CPT-AM, as well as other 8-CPT derivatives, have been reported to induce off-targets such as PDE activation, thus requiring caution when analyzing their effects. Recently, Yarwood’s group identified, in a high throughput screening (HTS) study, the first known non-cyclic nucleotide (NCN) EPAC1 agonist, I942 with in vitro application [32,33]. NMR spectrometry analysis demonstrated that I942 can stabilize an inhibition-incompetent activation intermediate state of EPAC1 distinct from both active and inactive states [34]. Additionally, in a study carried out by the Zhou’s group, a series of non-cyclic nucleotide activators named 25g, 25q, 25n, 25u, 25e, and 25f with good selectivity over PKA were identified. Among them, compounds 25e, 25f, 25n, and 25u show a higher selectivity over EPAC1. Specifically, the agonist 25u inhibits STAT3 activation, and subsequent pro-inflammatory IL-6 signaling to Vascular cell adhesion protein 1 (VCAM1) induction, showing a higher activity than its precursor I942 [35]. Another non-cyclic nucleotide, SY009 agonist was isolated by ultra HTS in a library of 350,000 compounds. SY009 is chemically distinct from I942 and displays higher selectivity for EPAC1 over EPAC2 and PKA [36]. Subsequent studies must be performed to investigate the functional effects of these compounds.

Many studies reported detrimental effects of EPAC1 activation during stress conditions [9,14]. Therefore, efforts have been made to identify potential EPAC1 inhibitory compounds. Pioneer studies characterized small molecules that prevented both cAMP-dependent EPAC1 and EPAC2 activation. Among these pan-EPAC inhibitors, ESI-08 and ESI-09 were identified from HTS as non-cyclic compounds that impede EPAC’s activity [37,38,39]. Interestingly, ESI-09 has demonstrated significant pharmacological effect in preventing the invasion and metastasis of pancreatic and breast cancers, as well as protecting from fatal rickettsioses bacterial infection [39,40]. Nonetheless, thermal shift assays showed that high concentrations (>25 μM) of ESI-09 had a detrimental effect on protein stability [41]. Optimization of ESI-09 led to different inhibitors from which NY0123 showed to be a more potent inhibitor than its precursor in vitro [42]. The above-mentioned compounds are classified as competitive inhibitors of EPAC1, but non-competitive and uncompetitive inhibitors have also been characterized [14]. In the last two categories, both types of compounds bind to an allosteric site of the protein. While a non-competitive inhibitor binds both to the protein in the absence or presence of the substrate, an uncompetitive inhibitor requires that the protein substrate complex is previously formed. Of note, uncompetitive inhibitors are more effective at increasing concentrations of substrate, which highlights their potential for therapeutics. Compound 5376753, for example, is a non-competitive EPAC1 and EPAC2 inhibitor that was found by a combination of computational analyses and a Bioluminescence Resonance Energy Transfer (BRET)-based assay called CAMYEL and inhibits Rap1 activation [43]. The highly selective and potent EPAC1 uncompetitive inhibitor, CE3F4 was identified in an HTS assay in 2012 [29]. CE3F4 blocks EPAC1 guanine nucleotide exchange activity toward its effector Rap1 both in cell-free systems and in intact cells [29,44]. CE3F4 has been successfully used in a multitude of studies to reveal EPAC1-dependent biological action in different cell types [14]. The selectivity of (R)-enantiomer of CE3F4(CE3F4R) for EPAC1 is determined by one amino acid of difference with EPAC2 (EPAC1: Q270; EPAC2: K405) [45]. The binding site for the CE3F4R is located in the CNB domain of EPAC1, at a specific position that is exposed when cAMP binds, thus forming an EPAC1-cAMP-CE3F4 ternary complex. CE3F4R interacts with EPAC1 in an intermediate state that stabilizes its closed topology, thus preventing EPAC1 catalytic activity [45,46]. More recently, Laudette and collaborators [47] isolated a thieno [2.3-b]pyridine derivative (called AM-001) that behaves as a non-competitive and selective EPAC1 inhibitor with no effect on EPAC2 or PKA activity. Interestingly, AM-001 displays in vivo applications and mitigates cardiac hypertrophy, inflammation, and fibrosis after chronic β-adrenergic activation in mice. In addition, acute injection of AM-001 is cardioprotective against myocardial ischemia/reperfusion (I/R) injury [48]. Recently, Buffano and collaborators used cosolvent molecular dynamics to determine the binding site of AM-001 [49]. They found that the allosteric site of AM-001 lied in a pocket formed by CDC25-HD and CNB domains of EPAC1. Binding of AM-001 to this pocket stabilized an inactive “like” state of EPAC1 by strengthening the interaction of these two domains [49].

## 4. EPAC Compartmentation

### 4.1. Integration of Membrane Signaling

GPCRs function as a link between extracellular signals and intracellular signaling routes and play a crucial role in the activation of EPAC1. In the heart, β-adrenergic (β-AR) receptors are among the most important GPCRs that enhance myocardial performance in response to stress or exercise. β-ARs are activated by catecholamines (adrenaline, noradrenaline) and exist as three subtypes in the heart, from which subtypes β1- and β2- ARs activate Gs proteins (stimulatory G proteins), leading to cAMP formation while β2 also signals through G_i/o_ (inhibitory G proteins) [50,51]. Although cardiac β3-AR has not been described in detail yet, it is coupled to G_i/o_ proteins and NOS-dependent production of cGMP [52]. Acute stimulation of β-ARs is beneficial for the heart, nevertheless, prolonged activation may cause cardiac remodeling including hypertrophy and fibrosis, which can evolve into heart failure [53]. Interestingly, EPAC1 is activated by both β1-AR and β2-AR that induce different biological responses; pro- and anti-hypertrophy, respectively [54]. Of note, EPAC1 is increased at the onset of cardiac hypertrophy in a PKA-independent manner and EPAC1 pharmacological or genetic inhibition protects mice against cardiac hypertrophy and fibrosis during chronic β-AR stimulation [48,55,56].

**Figure 2:**
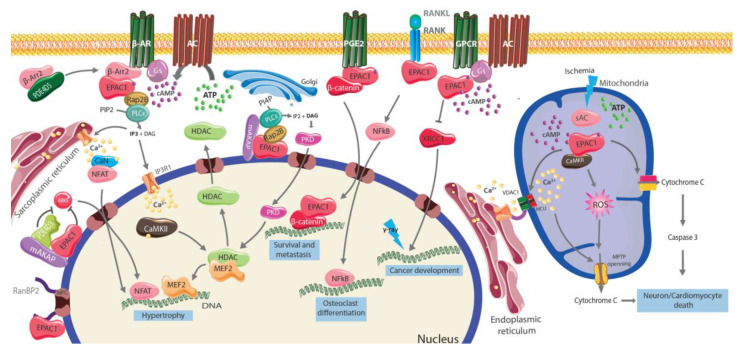
EPAC1 signalosomes. Upon activation of the β-AR, the β-arr2-EPAC1 complex is recruited to the plasma membrane where EPAC1 activates RAP2B and PLCε leading to IP3 production. This signaling pathway stimulates in turn calcineurin (CaN) and Ca^2+^/calmodulin-dependent protein kinase II (CaMKII). Once phosphorylated by CaN, the nuclear factor of activated T cells (NFAT) translocates to the nucleus to induce the transcriptional activation of prohypertrophic genes. CaMKII promotes the nuclear export of histone deacetylase (HDACs) and thus the activation of the prohypertrophic transcription factor, myocyte enhancer factor 2 (MEF2). PI4P produced in the Golgi activates the nuclear complex Epac1/PLCε/A-kinase anchoring protein (mAKAP) at the nuclear membrane leading to PKD activation and HDAC export.

Mechanistically (Figure 2), EPAC1 forms a molecular complex with the scaffold protein β-arrestin2 (β-arr2), when β1-AR is activated the complex is translocated to the plasma membrane. β-arr2 binds to the C-terminal part of the β1-AR allowing the activation of EPAC1 and subsequent cardiomyocyte hypertrophy [54,57]. Accordingly, β-arr2 knock-down prevented cardiomyocyte hypertrophy after activation of β1-AR with isoproterenol. It is important to highlight that the interaction of EPAC1 with β-arr2 depends also on the presence of the cAMP-specific PDE variant, PDE4D5. The latter interacts with β-arr2 hence inhibiting EPAC1 interaction necessary for its pro-hypertrophic effect [54]. Pro-hypertrophic signaling pathways of EPAC1 have been studied in depth [13]. The calcium-sensitive proteins, calcineurin, and calmodulin-dependent protein kinase II (CaMKII) act as key effectors of EPAC1 in its functional effect in cardiomyocytes [56,58,59,60,61]. Additional effectors have been shown to participate in this process. For instance, Morel and collaborators [62] showed that the pro-hypertrophic effect of EPAC1 involved the small GTPase, Rac. Moreover, EPAC1 has been shown to activate Rap2B-phospholipase C (PLC) and H-Ras leading to CaMKII activation [54] and it was shown that the activation of Ras through EPAC1 was dependent on PLC and IP3R [59,63].

Activation of EPAC1 by GPCRs can also lead to the import of β-catenin to the nucleus and the activation of a battery of genes involved in survival and metastasis. Besides, activated EPAC1 can inhibit the repair of γ-ray-induced DNA damage by the degradation of X-ray repair cross-complementing protein 1 (XRCC1). On the other side, the activation of EPAC1 through RANK leads to NFκB activation and to promote osteoclast differentiation.

In the mitochondria EPAC1 promotes cell death and is activated by the sACs to increase the production of reactive oxygen species (ROS). In addition, EPAC1 facilitates the transfer of calcium from the ER to the mitochondria via a macromolecular complex composed of voltage-dependent anion channel 1 (VDAC1), the chaperone glucose-regulated protein 75 (GRP75), and the inositol-1,4,5-trisphosphate (IP3) receptor 1 (IP3R1), and the mitochondrial Ca^2+^ uniporter (MCU). The increase of mitochondrial ROS and Ca^2+^ leads to the opening of the mitochondrial permeability transition pore (MPTP).

The transcription factor, NFAT (Nuclear Factor of Activated T Cells) is a downstream effector of calcineurin and mediates cardiac hypertrophy development. Upon its dephosphorylation by calcineurin, NFAT translocates to the nucleus to regulate its target genes [64]. Work from our laboratory shows that EPAC1 upregulates the expression of *MCIP1*, a well-known modulator of calcineurin signaling that possesses a series of NFAT response element in its promoter [62]. Besides, activation of EPAC1 leads to the upregulation and activation of Transient Receptor Potential Canonical (*TRPCs*) channels that sustain Ca^2+^ influx and calcineurin/NFAT activity [65]. Yet, the silencing of EPAC1 suppressed the translocation of NFATc3/c4 induced by Isoproterenol in guinea pig hearts [66]. Aside from NFAT, EPAC1 effects on gene transcription involve other players like myocyte enhancer factor 2 (MEF2), a transcription factor involved in hypertrophy development [67]. In this sense, CaMKII activation by EPAC1 regulates histone deacetylases 4 and 5 (HDAC) by inducing their export from the nucleus thus derepressing MEF2 and leading to pro-hypertrophic signaling [59,63]. Interestingly, recent work from our laboratory shows that EPAC1 interacts with the G protein-coupled receptor kinase 5 (GRK5) to promote cardiac myocyte hypertrophy. Indeed, GRK5 acts as a downstream mediator of EPAC1, allowing the phosphorylation of HDAC5 and subsequent MEF2 activation following activation of β-AR with isoproterenol [48].

It is worth mentioning that EPAC1-calcineurin/CaMKII signaling pathway is not limited to the heart and mediates various biological actions such as neuronal death (see below). In diabetic nephropathy, increased EPAC1 mRNA levels lead to cell cycle arrest and cellular hypertrophy leading to kidney tubular hypertrophy [68]. Additionally, EPAC1 is also an important mediator of osteoclasts maturation induced by cAMP in bone-marrow cells. Silencing of EPAC1 in vitro reduced RANKL-mediated NFATc1 expression and osteoclasts differentiation [69].

### 4.2. EPAC Presence and Functional Relevance on the Nucleus

The presence of EPAC1 at the perinuclear envelope has been observed in many different cell types, suggesting an important role in the regulation of nuclear function. Anchoring of EPAC1 at the cytosolic side of the nuclear pore complex (NPC) has been reported to be responsible for EPAC’s nuclear localization. By a two-hybrid screen, Gloetrich and collaborators [70] showed that EPAC1 interacted with a component of the NPC, RanBP2, through its zinc finger domain. This interaction was sufficient for its localization to the nuclear membrane (NM) but inhibited Rap1-dependent EPAC activity. In another study, mass spectrometry analyses identified additional proteins complexed to EPAC1 at the NM, such as Ran, importin β1, and nucleoporins 98 and 205 [71]. However, in contrast with the previous study, the association of EPAC1 with Ran was demonstrated to be essential for NM localization and this association facilitated Rap1 activation. In 2015, Parnell identified for the first time a sequence responsible for the nuclear localization of EPAC1 within the CDC25-HD domain (amino acids 764 to 838) (Figure 1D). This sequence does not correspond to a canonical nuclear localization signal. Hence, its deletion impedes EPAC1 nuclear localization in HEK293 cells [22]. Surprisingly, this Nuclear Pore Localization Signal (NPLS) is also conserved in EPAC2 that displays mainly cytosolic localization. With this in mind, the authors propose a model whereby the CNB1 domain of EPAC2 blocks the NPLS hence impeding the protein nuclear localization [22]. In a neuronal cell line, it has also been shown that the interaction of EPAC1 with Importin β1 is important for its localization to the NM. Disruption of this complex avoids the nuclear localization of EPAC1, favoring its accumulation in the plasma membrane where EPAC1 inhibits neurite outgrowth [72]. In adult cardiomyocytes, adenovirus transduction of EPAC1-GFP demonstrated sarcolemmal, and strong perinuclear localization [56]. Furthermore, the group of Bers [73] labeled cardiomyocytes isolated from wild type (WT), EPAC1 knock-out (KO), and EPAC2 KO mice with a fluorescent cAMP derivate EPAC ligand, 8-[Pharos-575]-2′-O-methyladenosine-3′,5′-cyclic monophosphate, to show that EPAC1 and EPAC2 had distinct subcellular distributions. EPAC2 was mainly found on the T tubules whereas EPAC1 was essentially observed in the nucleus [73].

Little is known to date about the exact function exerted by nuclear EPAC1, but growing evidence supports a role in the modulation of hypertrophy. Accordingly, Dogde-Kafka found that muscle-specific mAKAP assembled a cAMP-responsive network at the NM of cardiomyocytes with PDE4, PKA, and EPAC1 that finely tuned ERK5 activity through Rap1 to modulate hypertrophic response [74]. As an alternative mechanism, it was previously described that PLCε was an important downstream effector of cAMP/EPAC1 pathway through the action of Rap2B [75]. Zhang and collaborators [76,77] later demonstrated that PLCε was located at the NM of cardiomyocytes and formed a complex with AKAPβ and EPAC1 required for agonist-dependent hypertrophy. They elegantly showed that upon hypertrophic stimuli, PLCε downstream of EPAC1 generated diacylglycerol (DAG) from a PI4P pool in the perinuclear Golgi allowing activation of nuclear protein kinase D (PKD) and the release of nuclear Ca^2+^ necessary for HDAC5 nuclear export and stimulation of hypertrophic genes [77]. Besides hypertrophy, nuclear EPAC1 has been shown to regulate other cellular responses such as an epithelial-to-mesenchymal transition in lung carcinoma cells [78]. It is suggested that EPAC1 interacts with β-catenin, a protein that translocates to the nucleus upon prostaglandin E2 treatment and activates the transcription of a battery of genes involved in survival and metastasis. Interestingly, both EPAC1/β-catenin interaction and PGE2-mediated transcription were abolished with the EPAC1 mutant deleted for the NPLS sequence (amino acids 764 to 838), indicating a preferential role of EPAC1 at the nuclear membrane. In Hela cells, Houston and collaborators [79] showed that EPAC1 was capable of mediating DNA-dependent protein kinase (DNA-PK) translocation from nucleus to the cytosol, proposing a new role for EPAC1 in the regulation of DNA repair process and Akt kinase activation. While this mechanism has not been observed in other cells or tissues, future studies will be necessary to assess its relevance in pathophysiological conditions.

### 4.3. EPAC Presence and Functional Relevance in the Mitochondria

Cytosolic cAMP is unable to cross the inner mitochondrial membrane and therefore local production of cAMP in mitochondria is carried out by the sAC which is activated in response to HCO3- and Ca^2+^ [3]. Mitochondrial localization of EPAC1 was determined jointly with its nuclear localization by Cheng’s laboratory in COS-7 cells [15]. After this, Wang and collaborators [80] found that EPAC1 and EPAC2 were present in isolated cardiac mitochondria. At the same time, our laboratory employing subfractioning of mitochondria found that EPAC1 was present in the mitochondrial inner membrane and matrix [81]. The presence of EPAC1 at the mitochondria was confirmed by subcellular fractionation together with live-cell co-localization imaging in vascular smooth muscle cells (VSMC) [82]. A putative mitochondrial localization signal was proposed by Qiao in the N-terminal region of EPAC1 [15]. Our laboratory narrowed down this putative sequence to a smaller sequence (del2-37, Figure 1D) and showed that it was responsible for the targeting of EPAC1 to the mitochondria [81].

Mitochondria play a crucial role in cell death by regulating necrosis and apoptosis. Recent advances in the field suggest that these processes are not independent of one another but are linked both functionally and mechanistically [83]. Briefly, apoptosis involves the permeabilization of the outer membrane (MOMP) and this process is regulated by the B cell lymphoma-2 (BCL-2) family of proteins. Namely, BCL-2, BCL-2-associated X protein (BAX), BCL-2 antagonist/killer 1 (BAK), BCL-2-like 11 (BIM), BH3 interacting domain death agonist (BID), the p53-upregulated modulator of apoptosis (PUMA), BCL-2-associated agonist of cell death (BAD), among others. Activation of BAX and BAK following MOMPC allows the exit of Cytochrome C into the cytosol to form the apoptosome. In this way activation of Caspase-9, -3, and -7 leads to apoptosis. Necrosis pathways, on the other hand, can be fired by calcium overload, increased cellular reactive oxygen species (ROS), and adenine nucleotide depletion. All these processes ultimately lead to the opening of the mitochondrial permeability transition pore (MPTP) in the inner mitochondrial membrane (IMM). This generates the dissipation of the proton gradient in the membrane (depolarization) and cell death.

EPAC1’s role in cell death has been reported in different models with opposite implications. In the heart, glucagon-like peptide receptor (GLP-1R) activation attenuated oxidative stress in cardiomyocytes treated with H_2_O_2_ in an EPAC1-dependent manner through the upregulation of antioxidant enzymes. Furthermore, the authors showed that, upon GLP-1R activation, a synergistic effect of EPAC1 and PKA protected the cells from apoptosis by impeding caspase-3 activity and upregulating *Bcl-2* expression [84]. Accordingly, the work of Khaliulin and collaborators [85] found that activation of PKA and EPAC1 mediated the protection against I/R injury in rat hearts. It is noteworthy that simultaneous activations of EPAC1 and PKA were necessary to drive cardioprotection in the aforementioned studies. In addition, the precise localization of EPAC1 mediating these effects was not mentioned. In contrast, Wang and collaborators [80] showed that activation of sAC by HCO3-, which is specifically expressed in mitochondria, delayed transmembrane potential (ΔΨm) loss, Ca^2+^ entry, and MPTP opening. This effect was inhibited by the selective EPAC1 inhibitor CE3F4 [80]. Recent work from Jayarajan and collaborators [86] showed that sAC through EPAC1 controlled basal AMPK activity and thus supported mitochondrial clearance, cellular energy, and redox homeostasis in H9C2 cardiomyoblasts. In the kidney, activation of EPAC in proximal tubular epithelial cells showed a protective effect against cisplatin nephrotoxicity. The reduced apoptosis and the preservation of the cell-cell junction were dependent on EPAC and Rap activation [87].

The work reviewed so far favors the idea of a protective role for EPAC1, but work from our laboratory found that EPAC1 played a crucial role in inducing cell death in the heart. We found that EPAC1 KO mice subjected to myocardial I/R were protected from oxidative stress and cardiomyocyte apoptosis [48,81]. Consistently, genetic ablation of EPAC1 limited the upregulation of Bax and cytochrome C, the downregulation of Bcl2, and the activation of caspases 3 and 9. In a model of in vitro hypoxia-reoxygenation, activation of EPAC1 through sAC increased mitochondrial Ca^2+^ uptake and ROS production leading to MPTP opening and cell death [81]. Particularly, Fazal and collaborators generated a mutant that lacked the mitochondrial signal (EPAC1 Δ2-37) and was excluded from the mitochondria. This mutant did not impair Rap1 activation, meaning that the EPAC1 cytosolic function was intact. Upon hypoxia and reoxygenation, cardiomyocytes transfected with this mutant exhibited less cell death than the controls, thus confirming a crucial role of mitochondrial EPAC1 in cell death. The contradicting results to that of Wang can be explained by the different models used to induce cardiomyocyte apoptosis in distinct pathophysiological contexts. In line with our results, EPAC1 inhibitors (ESI-09 and CE3F4) limited the accumulation of Ca^2+^ in the mitochondria in the adrenocortical cell line (H295R) [88]. The Ca^2+^ accumulation induced by overexpression of a mitochondrial-targeted sAC was inhibited [88].

After aortic banding in WT and EPAC1 KO mice, the absence of EPAC led to reduced apoptosis measured as TUNEL positive cells and reduced BAX expression in comparison with WT mice [89]. A role for EPAC1 in modulating mitochondrial dynamics was proposed since its genetic ablation or pharmacological inhibition impeded mitochondrial fission [82].

In the nervous system, recent work from Liu’s laboratory found that EPAC1 deletion protected against retinal neuronal apoptosis and necroptosis after retinal I/R injury [8] In line with a neuroprotective effect of EPAC1 inhibition, Suzuki and colleagues found that stimulation of EPAC1 in cortical neurons increased apoptosis, Bax/BCL-2 ratio, and Bim/Bcl-2 interactions [90]. In addition, in vivo injection of an irreversible inhibitor of mitochondria complex II, 3-nitropropionic acid failed to promote neuronal apoptosis in EPAC1 KO mice [90]. These results suggest that EPAC1 induces neuron-specific apoptosis through increasing Bim expression.

In cancer also contradicting results have been shown in line with what we presented so far. In breast cancer cell lines, treatment with a pan-EPAC antagonist, ESI-09 prevents cell migration and induces apoptosis [91]. In contrast, the PDE4 inhibitor Rolipam aids A172 human glioblastoma cell line death through the activation of EPAC1 [92]. In lung cancer cells cAMP through EPAC action inhibits the repair of γ-ray-induced DNA damage by the degradation of X-ray repair cross-complementing *protein* 1 (XRCC1) [93]. However, these inconsistencies may be because different types of cancer cells can present different expression patterns and thus similar stimuli can have opposite outcomes.

## 5. Discussion

Although there are some conflicting results in the literature, EPAC1 has a proven effect on transcriptional regulation that plays an important role in cardiomyocyte hypertrophy. On the other side, in the mitochondria, EPAC1 would be crucial in the processes of cell death, both apoptosis, and necrosis, though further studies need to be carried out to understand the detailed mechanism. The information gathered in this review puts EPAC1 on a preferential place as a potential therapeutic target in cancer development, cardiovascular diseases, and neurodegenerative disorders, among others. In addition, although it is well known that age predisposes to the development of the aforementioned pathologies, very few studies have evaluated the potential role of EPAC1 in the process of aging itself. In line with this, the functions of EPAC1 identified in the different signalosomes are among the key factors that regulate aging. Namely, mitochondrial EPAC1 has been shown to regulate ROS, mitochondrial fission/fusion, and death pathways while nuclear EPAC1 modulates the localization of DNA repair enzymes. These results highlight the importance of studying the role of EPAC1 in aging. In the future, improved knowledge of the mechanisms of action of EPAC1 signalosomes will help to design original therapeutic tools for the treatment of various disorders.
